# Simultaneous EEG-fMRI: Evaluating the Effect of the EEG Cap-Cabling Configuration on the Gradient Artifact

**DOI:** 10.3389/fnins.2019.00690

**Published:** 2019-07-10

**Authors:** Muhammad E. H. Chowdhury, Amith Khandakar, Karen J. Mullinger, Nasser Al-Emadi, Richard Bowtell

**Affiliations:** ^1^Department of Electrical Engineering, College of Engineering, Qatar University, Doha, Qatar; ^2^Sir Peter Mansfield Imaging Centre, University of Nottingham, Nottingham, United Kingdom; ^3^Birmingham University Imaging Centre, School of Psychology, University of Birmingham, Birmingham, United Kingdom

**Keywords:** EEG artifact correction, EEG cap-cabling configuration, gradient artifact, ribbon cable, simultaneous EEG-fMRI

## Abstract

Electroencephalography (EEG) data recorded during simultaneous EEG-fMRI experiments are contaminated by large gradient artifacts (GA). The amplitude of the GA depends on the area of the wire loops formed by the EEG leads, as well as on the rate of switching of the magnetic field gradients, which are essential for MR imaging. Average artifact subtraction (AAS), the most commonly used method for GA correction, relies on the EEG amplifier having a large enough dynamic range to characterize the artifact voltages. Low-pass filtering (250 Hz cut-off) is generally used to attenuate the high-frequency voltage fluctuations of the GA, but even with this precaution channel saturation can occur, particularly during acquisition of high spatial resolution MRI data. Previous work has shown that the ribbon cable, used to connect the EEG cap and amplifier, makes a significant contribution to the GA, since the cable geometry produces large effective wire-loop areas. However, by appropriately connecting the wires of the ribbon cable to the EEG cap it should be possible to minimize the overall range and root mean square (RMS) amplitude of the GA by producing partial cancelation of the cap and cable contributions. Here by modifying the connections of the EEG cap to a 1 m ribbon cable we were able to reduce the range of the GA for a high-resolution coronal echo planar Imaging (EPI) acquisition by a factor of ∼ 1.6 and by a factor of ∼ 1.15 for a standard axial EPI acquisition. These changes could potentially be translated into a reduction in the required dynamic range, an increase in the EEG bandwidth or an increase in the achievable image resolution without saturation, all of which could be beneficially exploited in EEG-fMRI studies. The re-wiring could also prevent the system from saturating when small subject movements occur using the standard recording bandwidth.

## Introduction

Simultaneous Electroencephalography (EEG) and functional Magnetic Resonance Imaging (fMRI) has turned into a popular method in the study of neuronal signal (Andreou et al., 2017; Arichi et al., 2017; Brueggen et al., 2017; Feige et al., 2017; Pittau et al., 2017; Tsuchimoto et al., 2017). The combined technology with higher spatial resolution of fMRI and high temporal precision of EEG have enabled to unlock the prospects of emerging an improved understanding of the brain functionality and the underlying principle of the haemodynamic signal measured in fMRI (Mayhew et al., 2016; Mullinger et al., 2017; Tsuchimoto et al., 2017). Simultaneous EEG−fMRI has primarily been used to relate electrophysiological and haemodynamic measures of brain activity made during spontaneous changes in brain state (i) at rest (Laufs et al., 2003), (ii) during sleep (Wilson et al., 2015) or (iii) due to pathology, such as epilepsy (Masterton et al., 2013); or in single−trial responses to sensory, motor or cognitive tasks (Mullinger et al., 2014). This has provided new insight into the origin of neural oscillations (Scheeringa et al., 2016), the origin of haemodynamic responses and the role of neurovascular coupling (Andreou et al., 2017; Arichi et al., 2017; Brueggen et al., 2017; Feige et al., 2017; Mullinger et al., 2017). In addition, it has been shown that simultaneous EEG−fMRI can provide greater specificity regarding the temporal sequence (Mayhew et al., 2012; Pisauro et al., 2017; Pittau et al., 2017) of activity in responsive brain areas, compared with that provided by standard analysis of single−modality neuroimaging data. Although a significant advancement has been made recently on simultaneously acquiring EEG and fMRI, but the quality of recorded EEG signals inside the MR scanner still requires considerable improvement.

The recorded EEG signal during the concurrent EEG-fMRI are compromised by several artifacts, which can overwhelm the actual brain signal. Allen et al. (2000) showed that the artifact from the time-varying magnetic field gradients used for the MR imaging is the most significant artifact. This induce voltages in the leads of the EEG system as well as in the head volume conductor (Chowdhury et al., 2019). The resulting gradient artifact (GA) is found to be three folds stronger than the weak brain signals (Mullinger et al., 2011). The stronger the gradient artifacts, the greater the efforts needed to overcome them to extract the weak neuronal signals using artifact correction during post-processing. Any small residual GA can simply swamp the neuronal signals of the brain. However, Allen et al. (2000) showed a template based average artifact subtraction (AAS) technique for GA correction, which become very popular among the researchers. In AAS, an average GA template is extracted and then it is subtracted from each occurrence of the GA. It needs precise sampling of the GA waveform in each occurrence, and the magnitude of the artifact must be smaller than the dynamic range of the EEG amplifier. The necessity of precise sampling issue can be solved by synchronizing the EEG system and MR scanner clocks while setting the slice-repetition time (TR) as an integer multiplication of the EEG period (Mandelkow et al., 2006; Mullinger et al., 2008). The non-saturated GA signals have to be acquired with an EEG amplifier with large dynamic range along with a hardware filter. However, the efficacy of the AAS compromises if the subject moves during the EEG data acquisition because of the alteration of the morphology of the induced GA (Mullinger et al., 2008, Eichele et al., 2010).

Without hardware filtering, the GA induced on the EEG leads can exceed 100 mV in magnitude easily, while the magnitude of the neuronal voltages is in the order of μV, which leads to the requirement of large dynamic range of EEG amplifier and a higher number of bits for digitization (Mullinger et al., 2011). However, a hardware low-pass filter can reduce the GA without removing the brain signals, because the major contribution of the GA power spectrum is contributed by the frequencies much higher than the brain signals. The cut-off frequency of the filter is normally set to 250 Hz in most of the studies as the main contribution of neuronal activities lies in the lower frequency band (Mullinger et al., 2008). This filter reduces the GA by a factor of ten or more to ensure the requirement of lower dynamic range of the EEG amplifier while allowing higher resolution analog to digital conversion in the EEG amplifier.

However, filtering the EEG data with lower cut-off frequency negatively effects the sampling accuracy of the artifact and also diminishes the opportunity of studying the neuronal activity at ultra-high band (Allen et al., 2000). It is also observed that in the presence of the hardware filter at some circumstances, some EEG channels are still saturated. Thus, it prevents artifacts correction, and more importantly, the amplifier saturation problem is still remaining. The situation can be even worse in the development of high-performance gradient systems in the future. It should be noted that reducing the GA magnitude at source could be useful in increasing the acquisition bandwidth of the EEG amplifier, which will consequently help to acquire and investigate EEG signal at higher frequency without saturating the EEG amplifiers.

Based on the above discussion, it is reasonable to reduce the EEG artifacts at source (Chowdhury, 2014). Researchers have proposed several approaches to reduce overall EEG artifacts from the raw data either at source or through post-processing. Among these, Reference Layer Artifact Subtraction (Chowdhury et al., 2014, 2019), Reference layer with standard EEG cap (Luo et al., 2014) and Reference layer adaptive filtering (Steyrl et al., 2017) had introduced the concept of using reference signals in the artifact correction; whereas Van der Meer et al. (2010) presented a carbon-wire loop based additional sensors and Abreu et al. (2018) proposed a family of methods using independent component analysis (ICA) for pulse artifact correction. GA correction was performed by Luo et al. (2014) in the concurrent EEG and fMRI acquisition through the calculation of artifactual template, which is modulated by the subject head position information and slowly changing splines.

**FIGURE 1:**
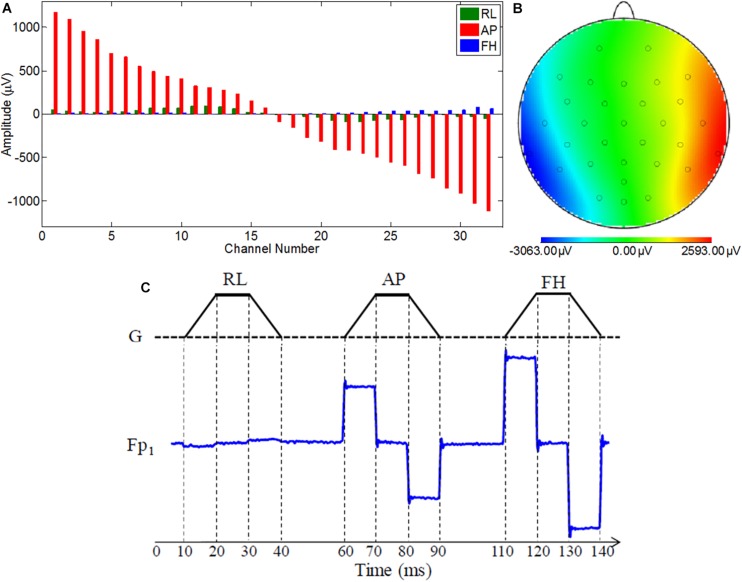
**(A)** Variation over channels of voltage recorded using the ribbon cable on application of the orthogonal gradients changing at 2 Tm^–1^s^–1^ (reproduced from Chowdhury et al., 2015). **(B)** Map of the artifact voltages induced on the EEG cap (excluding ribbon cable) on a subject’s head when an AP gradient changing at 2 Tm^–1^s^–1^ is applied. **(C)** Depiction of the customized EPI sequence to characterize the GA induced along each axis on an example lead for the ribbon cable.

However, new methods for reducing the unpredictability of the GA and its amplitude at origin using the existing EEG hardware are still very appealing to the researchers. Previous works (Yan et al., 2009; Chowdhury et al., 2015) have shown that the cable that links the EEG cap with the amplifier is responsible for considerable amount of GA under some circumstances. This is particularly the case for the flat ribbon cable, which is currently used by one popular MR-compatible EEG system manufacturer to connect the EEG amplifier to the EEG cap (Chowdhury et al., 2015). Figure 1A shows the voltages induced on the different leads in a 1 m long ribbon cable by time-varying gradients of 2 Tm^–1^s^–1^ magnitude applied along the three orthogonal gradients [i.e., right-left (RL), anterior-posterior (AP), and foot-head (FH) directions]. These induced voltages were measured while the ribbon cable was laid axially along the center of the MR scanner bore and its surface was perpendicular to the AP direction. This slow slew rate was used to characterize the GA induced in the EEG signal due to each gradient because of the limitation of the EEG amplifier bandwidth. Even with this slow slew rate, standard EEG acquisition bandwidth (0.016 – 250 Hz) was not able to well-characterized GA rather a higher acquisition bandwidth (0.016 – 1000 Hz) had to use for EEG data acquisition.

**FIGURE 2:**
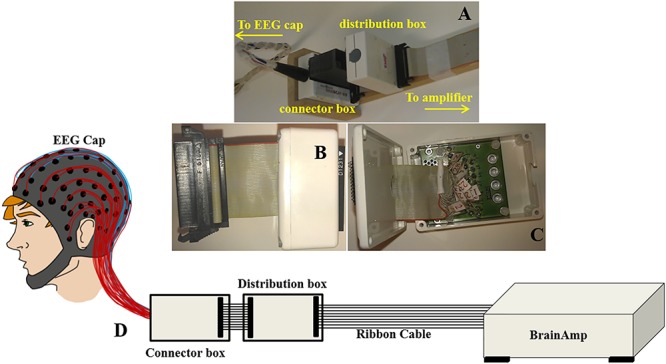
Images displaying **(A)** the ribbon cable attachment to the cantilever and connection to the distribution box (standard/modified) and the connector box of the EEG cap (reproduced from Chowdhury et al., 2015); **(B)** the distribution box and **(C)** wiring inside the distribution box; **(D)** Schematic representation to show out-of-the-box configuration of BrainAmp Cap-cabling and re-wiring module, ribbon cable, and amplifier.

With this configuration, the largest induced voltages in the leads are due to the AP gradient (Chowdhury et al., 2015), and the voltages due to this gradient increase linearly with the increase of lead number. This corresponds to the variation in the effective loop area created by each lead in the ribbon cable and the central lead which corresponds to the reference channel (Chowdhury et al., 2015). The magnitude of these voltages, which vary from −1121 to 1173 μV, is comparable to the magnitude of the induced voltages for the head and EEG cap by a similar temporally varying gradient. The results for an AP gradient varying at 2 Tm^–1^s^–1^ were shown as a spatial map of the head in Figure 1B. These voltages were measured by connecting the EEG cap (mounted on human head) and the EEG amplifier through a twisted cable in place of the ribbon cable, where the cable contribution is negligible (Chowdhury, 2014; Chowdhury et al., 2015). It is clear from Figure 1B that the GA generated in the EEG cap and the ribbon cable is dependent on how the EEG cap is made, the position of a particular lead in the ribbon cable and also on the location of the electrode on the cap. Since in current practice this correspondence is set arbitrarily, it is important to set the electrodes and leads configuration in an optimal way which can reduce the GA at source. This approach could reduce the highest induced voltage recorded by the EEG amplifier and thereby reduce the requirement of higher dynamic range of the EEG amplifier. The main objective of this paper is to quantify the effect of cap-cable configuration on the characteristics of the GA, which could be used to identify an optimal wiring arrangement to reduce the gradient artifact induced in the EEG data in the simultaneous EEG-fMRI experiment. In this work, we studied two different cap-cable configurations: (1) A 1m long ribbon cable attached to a distribution box (Figure 2A) with standard wiring configuration, effectively connecting the EEG amplifier to the cap without altering the standard wire and cable configuration; (2) The same ribbon cable attached to another distribution box with a modified wiring configuration chosen to minimize the induced artifact. It should be noted that the distribution box was added between the standard connection box (cap-cable termination) and the ribbon cable (connected to EEG amplifier).

Several experiments were carried out to evaluate the effectiveness of the modified configuration. Firstly, a study was carried out for both configurations (standard and modified) to measure the GA contribution for each of the orthogonal gradients (RL, AP, and FH) while a customized echo planar imaging (EPI) sequence (Mullinger et al., 2011) was applied sequentially along the three gradient axes. Another study was to evaluate the consequence of each cap-cable configuration on the GA voltages generated during the execution of low and high-resolution EPI sequences. The rest of the paper is organized as follows: Section “Materials and Methods” introduces how the re-wiring was chosen and implemented along with the experimental setup; Section “Analysis” describes the analysis technique followed by the results in Section “Results.” Section “Discussion” discusses the findings and finally the conclusion is provided in Section “Conclusion.”

## Materials and Methods

The setup of the experiment was similar to the setup used for our previous works (Chowdhury et al., 2015, 2018). EEG signals were acquired inside a 3 T Philips Achieva MR scanner (Philips Medical Systems, Best, Netherlands) using a standard EEG cap (32-electrode), and BrainAmp MR-plus EEG amplifier using the BrainVision EEG Recorder software (Brain Products, Munich, Germany). The sampling rate of the EEG signal was set to 5 kHz to make sure the EEG amplifier and MR scanner clocks were synchronized and precise sampling of EEG and GA waveforms can be obtained (Mandelkow et al., 2006; Mullinger et al., 2008). Slice acquisition trigger pulses from the MR scanner were applied to the EEG system for this synchronization.

A ribbon cable and a twisted cable of one-meter length were mounted on a wooden beam running axially along the magnet bore and placed on a wooden stand behind the head-end of the MR scanner. The wooden stand was kept on the floor to confirm that it was held straight and not affected from the vibration of the MR scanner (Mullinger et al., 2011; Chowdhury et al., 2018). The ribbon cable was kept in a manner so that the cable surface was parallel to the FH direction and the distribution box (standard/modified) and the connector box of the EEG cap were oriented normal to the FH direction (Figure 2). The EEG amplifier was kept outside the magnet bore on a wooden stand to isolate it from the scanner vibrations (Chowdhury et al., 2018).

### Optimizing the Connection Between EEG Cap and Amplifier

In the current wiring set-up of EEG cap to the amplifier, the EEG cap-cable bundle terminated in a connector box and the connection box is connected to the amplifier through the flat ribbon cable. There are two components of the wiring system which can contribute to GA artifact. First component was arising from the cap with cable bundle terminating at connection box and the second component was from the ribbon cable. In the current practice, the channels of ribbon cable arbitrarily connected to the cap-cable bundle channels and therefore overall GA artifact recorded from the set-up was not optimum. Table 1 shows the variation of GA artifact voltages induced on the EEG cap alone on a subject’s head. If the GA voltage induced in the EEG cap were sorted to produce largest positive to largest negative voltages to compare with the largest negative to positive voltage recorded using the ribbon cable when an AP gradient changing at 2 Tm^–1^s^–1^ was applied. For example, as shown in Figure 1A, the highest positive (negative) contribution from the ribbon cable were originating from channels 1–5 (27–31) due to the AP gradient while the largest negative (positive) contribution from the same gradient interacting with the EEG cap alone were produced on the right (left) side of the head (Figure 1B), thus channels 1–5 (27–31) of the ribbon cable can be connected to the right (left) side electrodes of the EEG cap to minimize the GA voltages generated from the AP gradient. Moreover, Table 1 shows that overall induced EEG voltage for AP gradient in individual channels can be minimized by connecting 1 to 18 EEG cap channels with 32 to 15 ribbon cable channels and 19 to 32 EEG cap channels with 14 to 1 ribbon cable channels, respectively. However, the electrocardiogram (ECG) and electrooculogram (EOG) channels of the EEG cap typically varies from trial to trial and subject to subject and therefore the contribution of these electrodes should not consider for re-wiring. In Table 2, it is shown that GA contribution from the EEG cap were sorted without ECG and EOG channels and their corresponding EEG channels name and number were tabulated to show which ribbon cable channels should be connected to cap-cable bundle channels to get the optimized re-wiring for AP gradient.

**TABLE 1:** Variation over channels of artifact voltages induced on the EEG cap (excluding ribbon cable) on a subject’s head and voltage recorded using the ribbon cable when an AP gradient changing at 2 Tm^–1^s^–1^ is applied.

**Channel No. of a EEG cap**	**Average AP amplitude in cap (μV)**	**Average amplitude in cap (μV) (sorted)**	**Channel No. of EEG cap (sorted)**	**Channel name of EEG Cap**	**Ribbon cable Channel No.**	**Average AP amplitude (μV)**
1	477	922	15	P7	32	−2100
2	−25	900	3	F3	31	−2006
3	900	889	32	ECG	30	−1820
4	−275	869	11	F7	29	−1720
5	867	867	5	C3	28	−1533
6	−318	729	7	P3	27	−1445
7	729	716	13	T7	26	−1258
8	125	646	25	FC5	25	−1159
9	467	547	20	Oz	24	−978
10	291	497	27	CP5	23	−892
11	869	477	1	Fp1	22	−702
12	−609	467	9	O1	21	−605
13	716	462	29	TP9	20	−418
14	−128	348	21	FC1	19	−330
15	922	343	23	CP1	18	−142
16	−54	291	10	O2	17	−44
17	−52	171	19	Pz	16	146
18	−9	125	8	P4	15	231
19	171	−9	18	Cz	14	417
20	547	−25	2	Fp2	13	516
21	348	−46	31	EoG	12	701
22	−231	−52	17	Fz	11	791
23	343	−54	16	P8	10	974
24	−84	−84	24	CP2	9	1072
25	646	−128	14	T8	8	1259
26	−775	−203	30	TP10	7	1347
27	497	−231	22	FC2	6	1533
28	−269	−269	28	CP6	5	1631
29	462	−275	4	F4	4	1818
30	−203	−318	6	C4	3	1908

*Sorting the artifact voltage (largest positive to largest negative) induced in the EEG cap (excluding ribbon cable) and comparing it with the induced voltages in ribbon cable arranging channels with largest negative voltage channels to largest positive voltage channel. It can be seen that connecting 1 to 18 EEG cap channels with 32 to 15 ribbon cable channels and 19 to 32 EEG cap channels with 14 to 1 ribbon cable channels to minimize overall induced EEG signal in individual channels.*

**TABLE 2:** Modifying Table 1 to exclude ECG and EOG channels in the re-wiring connection scheme as their nature can be unpredictable.

**Channel No. of EEG cap**	**Average AP amplitude in cap (μV)**	**Average amplitude (sorted) (μV)**	**Channel No. of EEG cap (sorted)**	**Channel name of EEG Cap**	**Ribbon cable channel No.**
1	477	922	15	P7	32
2	−25	900	3	F3	31
3	900	869	11	F7	30
4	−275	867	5	C3	29
5	867	729	7	P3	28
6	−318	716	13	T7	27
7	729	646	25	FC5	26
8	125	547	20	Oz	25
9	467	497	27	CP5	24
10	291	477	1	Fpl	23
11	869	467	9	O1	22
12	−609	462	29	TP9	21
13	716	348	21	FC1	20
14	−128	343	23	CP1	19
15	922	291	10	O2	18
16	−54	171	19	Pz	17
17	−52	889	32	ECG	16
18	−9	−46	31	EoG	15
19	171	125	8	P4	14
20	547	−9	18	Cz	13
21	348	−25	2	Fp2	12
22	−231	−52	17	Fz	11
23	343	−54	16	P8	10
24	−84	−84	24	CP2	9
25	646	−128	14	T8	8
26	−775	−203	30	TP10	7
27	497	−231	22	FC2	6
28	−269	−269	28	CP6	5
29	462	−275	4	F4	4
30	−203	−318	6	C4	3
31	−46	−609	12	F8	2

*This table summarizes the EEG cap channel name and number along with corresponding ribbon cable channel numbers for modified wiring.*

This modified wiring could be done in the connection box (Figure 2A) which will make this alteration as a permanent alteration and produce a risk of damaging the original EEG cap. Therefore, an additional box (distribution box) was attached to the path as shown in Figures 2A–D, to introduce this modification without changing the wiring in the original EEG cap connection box. Figure 2A shows how the ribbon cable and cap-cable bundle termination box, connector box is connected together with an additional box, distribution box. Figures 2B,C show the external and internal view of the distribution box. Figure 2D schematically shows how the distribution box placed between the connection box and ribbon cable to produce the re-wiring in-effect. Two distribution boxes were made for this work as discussed earlier, one with standard connection and other with modified connection.

### Experiments

A series of experiments were conducted to identify this optimal wiring arrangement to reduce the gradient artifact induced in the EEG data in the simultaneous EEG-fMRI experiment. A 32-channel EEG cap, similar to the one used by the author in the previous studies (Chowdhury et al., 2015, 2018), was used in the studies of this work. In this cap, there are thirty (30) electrodes were arranged according to the extended international 10–20 system and the location of the reference electrode was FCz (Chowdhury et al., 2015, 2018). An EOG channel was attached under the left eye of the subject and ECG channel attached to acquire ECG data for pulse artifact correction. EEG signals were acquired when the EEG cap was placed on the subject’s head while the Fp1 and Fp2 electrodes were placed axially at iso-center. To reduce the electrical skin contact impedance and increase signal-to-noise ratio, an Abralyte conductive gel was applied in each electrode location between the electrode and the scalp. EEG data were acquired on six healthy volunteers (age range: 20 to 35 years, mean: 27 years) during the execution of modified EPI sequence (Study 1) and three different multi-slice EPI sequences (Study 2) as shown in Table 3. All experiments were carried out at the Sir Peter Mansfield Imaging Centre (SPMIC), University of Nottingham, Nottingham, United Kingdom. Experiments on human subjects were carried out with the written consent from the subjects and with the approval from local ethical committee (ethical committee of the University of Nottingham).

**TABLE 3:** Details of the Multi-Slice EPI Sequences used for Study 2(i–iii).

		**Details**					
**Exp. No.**	**Multi-slice EPI sequences**	**TR (s)**	**TE (ms)**	**Matrix**	**In plane resolution (mm^2^)**	**Slice thickness (mm)**	**Phase-encoding direction**
(i)	Standard axial slice	2	40	80 × 80	3 × 3	3	AP
(ii)	High resolution coronal slice	2	40	160 × 120	1.5 × 1.5	0.5	RL
(iii)	Axial slice	2	40	60 × 60	4 × 4	4	RL

*Slice selection gradients were applied in FH in study 2(i) and (iii), and in AP direction in Study 2(ii).*

#### Study 1: Orthogonal Gradients

In order to understand the effect of the cap-cable configurations on the induced GA from the three gradients, a customized EPI sequence was deployed with the gradient pulses at a rate of 2 Tm^–1^s^–1^ one after the other in the RL, AP, and FH directions. EEG recordings were done during the execution of this customized EPI sequence (Mullinger et al., 2011). For this study, the EPI sequence was repeated for 30 times and the acquisition bandwidth for EEG amplifier was set to 0.016–1000 Hz with a 30 dB/octave roll-off to allow full characterization of the GAs and ADC resolution was set to 10 uV to obtain highest measurement range of ± 327.68 mV (Chowdhury et al., 2018). In this experiment, firstly, the cabling was terminated using the EEG cap on the human subjects, to test the interaction of the artifacts induced on the ribbon cable (with standard and modified connections) and later by a twisted cable to acquire the GA produced by the EEG cap alone (Chowdhury et al., 2015). This allowed measurement of the GA contributions of the elements of the system due to each orthogonal gradient before and after the modification of the ribbon cable connections.

#### Study 2: EPI

Twenty slices (with SENSE factor = 2, i.e., a two-fold decrease in the k-space lines) were acquired with equal temporal spacing for each TR-period. This results in slice acquisition rate of 10 slices/s for all experiments. In Study 2(i), use of a standard axial EPI sequence allowed to study the effect of the cap-cable configurations (as in Study 1) on the induced GA, which is conventionally used for whole brain fMRI study. In Study 2(ii), we used a high-resolution coronal slice acquisition which is commonly employed in fMRI of the visual cortex. In this case, the AP (slice) gradient makes the dominant contribution to the measured GA. Since the cap-cable optimization was done based on the contribution of AP gradient, it was expected that the modified wiring will have positive effect over standard wiring in this high-resolution coronal slice acquisition experiment. EEG data were acquired with a frequency range of 0.016–250 Hz over 30 volume acquisitions [Study 2(i) and Study 2(ii)]. This bandwidth is typically used in EEG-fMRI experiments to avoid saturation of EEG amplifiers and ADC resolution and measurement range were set to 0.5 uV and ± 16.384 mV, respectively. However, in Study 2(iii), EEG data were acquired with a frequency range of 0.016–1000 Hz to evaluate the cap-cable configuration at the higher recording bandwidth. Table 3 summarizes the fMRI parameters for all of these experiments in Study 2. It is important to note that the benefits of the modified wiring-configuration could be more pronounced for the standard EPI acquisition while readout gradient was in AP direction and phase-encoding gradient was in RL direction and the EEG signals were acquired with the increased bandwidth (0.016–1000 Hz).

## Analysis

EEG data were initially processed by the BrainVision Analyzer 2 (Version 2.0.1; Brain Products, Munich, Germany) and were further processed by MATLAB (The MathWorks).

### Study 1

The artifact induced in each channel was measured using the technique described in the author’s previous works (Chowdhury et al., 2015, 2018). In brief, the induced GA by each of the pulses (as shown in Figure 1C) was calculated over the flat 5 ms period after each 10 ms ramp period and averaged over 30 repetitions. The induced GA was quantified by calculating the difference of the induced voltages during the execution of ramp-up and down periods for each of the pulses. This help to remove the EEG baseline drift along with the high-frequency variations (Mullinger et al., 2011). The amplitude of the GA for each configuration and gradient direction was characterized by computing the range and the root-mean-square (RMS) of the EEG signal measured across electrodes and over subjects.

### Study 2

EEG data were exported from Brain Vision Analyzer 2 to MATLAB for analysis and the raw data (without down sampling or filtering) was analyzed so that the GA voltages over the entire frequency range are available for evaluation. In order to quantify the consequence of the cap-cable configuration for each EPI sequence, the artifact waveforms for each slice acquisition were baseline corrected with respect to the average 100 ms slice period. Then the mean and the RMS amplitude of the average artifact for each lead was calculated over the slice acquisition period. Mean and standard deviation over subjects for the different recordings were calculated. To assess the significance of the differences between the induced GA by the EPI sequences for the standard and modified cap-cable configurations, a paired *t*-test was accomplished on the data acquired from the studies.

In order to make sure that no substantial movements of the subject head were occurred during the experiments, fMRI data were realigned using SPM8. The RMS of the mean translational (x, y, and z) and rotational (pitch, yaw, and roll) realignment parameters were computed for each recording. The mean and standard deviation of the RMS realignment parameters over subjects was found. The RMS realignment parameters were compared to check whether any noteworthy non-voluntary motion was done by the subjects during the EEG-fMRI data acquisition during modified configuration but was absent in the standard configuration and vice versa.

## Results

### Study 1

**FIGURE 3:**
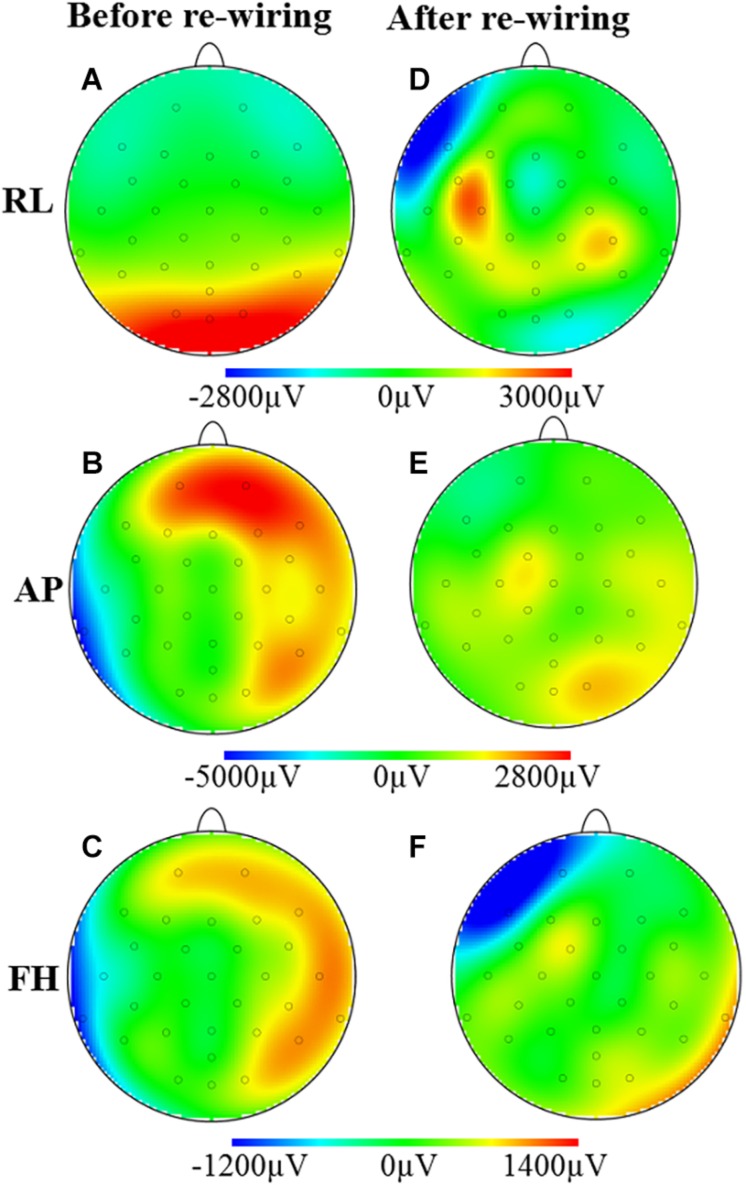
Maps of the artifacts induced by the RL, AP, and FH gradients while the EEG cap on the subject’s head: before **(A–C)** and after **(D–F)** re-wiring of the cap-cable configuration. This is showing the overall GA artifact alteration due to the re-wiring of the ribbon cable connected in between EEG cap and amplifier.

Figure 3 shows the spatial map of the RMS of the induced artifact voltage for the different orthogonal gradients for the different cap-cable configurations. It was observed that the modified configuration generated lower induced GA voltage from the AP and FH gradients compared with the standard configuration. The negative voltages were reduced more than the positive voltages (62/−30% compared with 31/23%) as a consequence of the non-linear distribution of voltages induced on the EEG cap due to the slight differences in lead paths for different channels.

Figures 3A,D demonstrates that the modified cap-cabling configuration greatly decreases the GA amplitude due to the RL gradient for O1, O2, Oz, and POz electrodes, but considerably increases the GA for F7, C3, FC5, CP5, and CP6 electrodes. The modified configuration substantially decreases the GA amplitude due to the AP gradient (Figures 3B,E). Figures 3C,F show the substantial reduction of the GA amplitude due to the FH gradient over the electrodes T7, TP9, P8, CP6, T8, F8, and Fp2, respectively. However, there is a substantial increase of the GA amplitude due to the FH gradient occurs at the Fp1 and F7 electrodes for the modified configuration.

**FIGURE 4:**
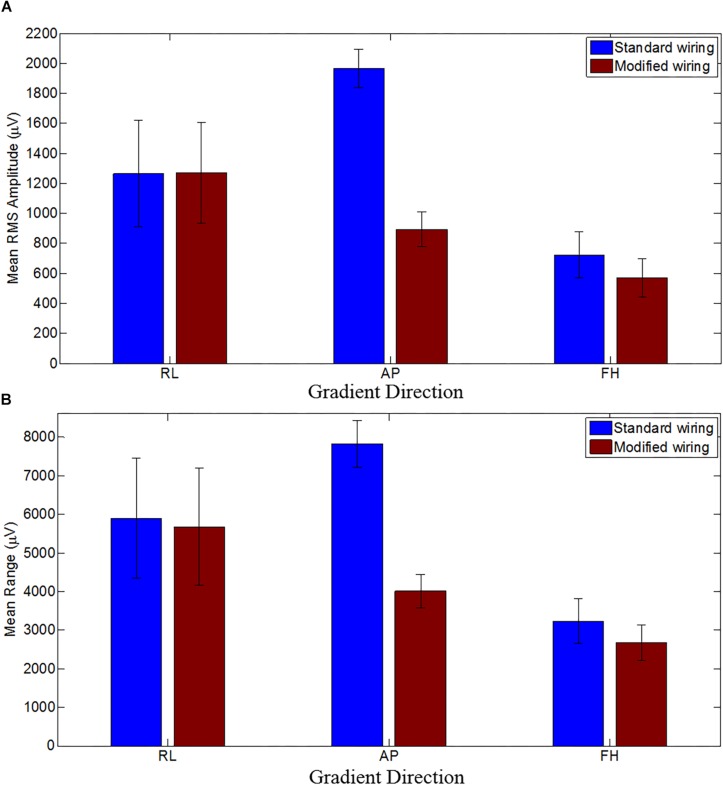
The mean RMS **(A)** and the mean range **(B)** of the induced GA over subjects for RL, AP and FH gradients for the standard (blue) and modified (brown) cap-cable configurations. Standard deviation over the subjects were shown by the error bars. Negligible difference with wide variation for RL gradients while considerable difference with small variations for AP and FH gradients over trials were observed.

Figure 4 shows the mean RMS and range of the GA induced over the subjects for the three different orthogonal gradients. When compared with the standard configuration, the modified cap-cable configuration shows 4, 49, and 17% reduction in range of the GA for the RL, AP, and FH gradients, respectively. There was a significant reduction (*p* < 0.0005) of the RMS GA amplitude were found for the AP (55%) and FH (21%) gradient, respectively. However, no notable change (*p* > 0.5) for the RL gradient was observed.

The average RMS GA amplitudes (across leads and subjects) before/after modifying the cap-cabling were found to be 1265 ± 354/1271 ± 339, 1963 ± 127/893 ± 117, and 725 ± 153/570 ± 129 μV, for RL, AP, and FH gradients, respectively. This indicates that the induced GA was reduced significantly (*p* < 0.0005 and *p* < 0.05) for the AP and FH gradient after changing the cap-cable configuration whereas there was no significant (*p* < 0.5) change in the GA induced by the RL gradient. There was a considerable variation (± 354/ ± 339) observed in this measure across subjects for the RL gradient. Table 4 shows the channel-wise GA artifacts for different gradients before and after re-wiring and their pair-wise comparison to show what extent the largest (positive/negative) artifacts were attenuated by the wring configuration. It was apparent from the Table 4 that while no considerable variation was observed for RL gradient, the re-wiring considerably reduces the GA contribution for the AP and FH gradients.

**TABLE 4:** Channel-wise GA artifacts for different gradients before and after re-wiring.

**RL**		**AP**		**FH**	
**Standard**	**Modified**	**Standard**	**Modified**	**Standard**	**Modified**
2983	2935	2811	1943	1323	1025
2845	2728	2782	1237	969	887
2579	2627	2449	1205	949	792
1694	1784	2268	669	938	699
1514	1599	2180	665	887	694
1290	1485	2046	657	870	648
1153	1074	2029	632	865	579
1058	1070	1781	522	848	543
1052	1029	1695	512	840	536
1022	958	1389	498	795	459
801	857	1099	474	506	427
791	834	636	302	499	401
594	816	624	221	480	389
517	676	96	160	381	363
431	536	75	149	371	251
359	455	−118	135	280	234
327	296	−146	114	270	214
295	176	−186	66	165	172
240	148	−324	21	154	170
−17	139	−515	−51	149	146
−22	118	−542	−305	97	107
−177	−116	−801	−425	72	33
−236	−211	−936	−449	65	21
−335	−252	−1040	−462	42	21
−378	−253	−1254	−493	−59	−4
−404	−478	−1410	−641	−77	−14
−498	−598	−1814	−747	−270	−24
−570	−682	−2287	−774	−297	−46
−826	−854	−2452	−850	−382	−182
−863	−906	−2982	−1472	−527	−193
−916	−1033	−4005	−1798	−1128	−703
−2869	−2721	−4906	−1874	−1804	−1457

This change confirms that the most consistent performance gain from the use of the modified configuration were produced for the AP and FH gradients. It was observed that the reduction of the GA for these gradients was less affected by small changes in position between repeated recordings on different subjects. The average range of GA amplitudes (across leads and subjects) before/after modifying the cap-cabling were found to be 5899 ± 1551/5679 ± 1513, 1963 ± 601/893 ± 117, and 3229 ± 581/2673 ± 460 μV, for the RL, AP, and FH gradients, respectively. A significant variation (*p* < 0.005) in the range of the induced GA voltage was observed for all the three applied gradients, when cap-cable configurations were changed. The average RMS and range of the GA amplitude (across leads and subjects) using the twisted cable (Chowdhury et al., 2015) in comparison to the ribbon cable were found to be 1245 ± 346/5809 ± 1555, 1277 ± 289/5711 ± 818 and 789 ± 85/3212 ± 596 μV, for the RL, AP, and FH gradients, respectively. Therefore, the modified ribbon cable configuration also outperforms the standard configuration with twisted cable in reducing the RMS and range of the induced GA. It can be noted that the modified cap-cabling configuration shown in this work out-performs the cabling configuration shown in Chowdhury et al. (2015).

### Study 2

**FIGURE 5:**
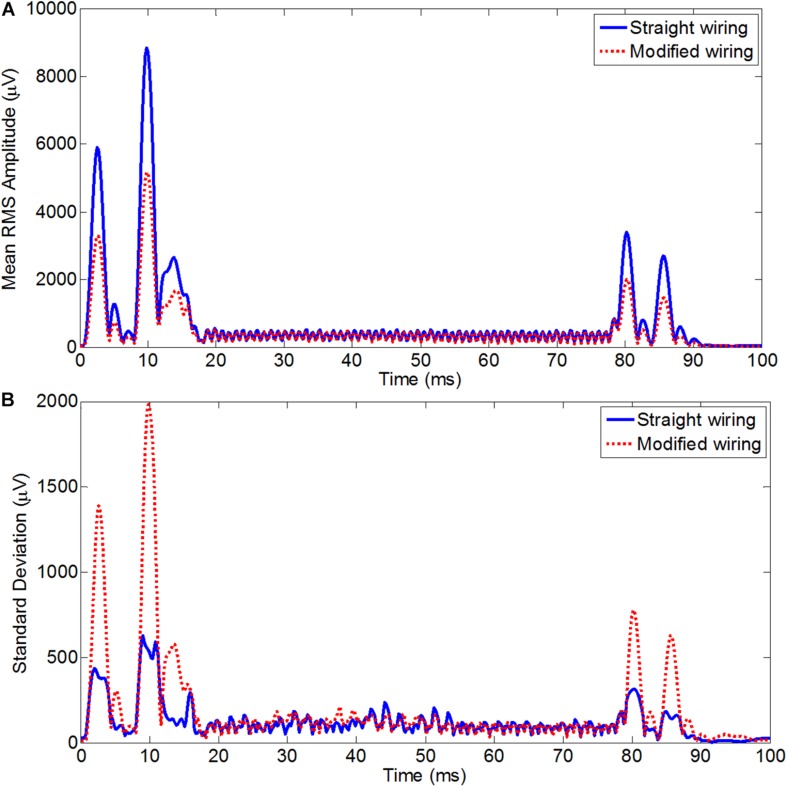
Average RMS across leads of the average slice artifact before (blue) and after (red) re-wiring for axial EPI slice acquisition **(A)** and for coronal EPI slice acquisition **(B)** across all subjects. This experiment should be particularly advantageous for high resolution coronal acquisition as typically used for visual cortex experiments.

Figure 5 shows the RMS of the average induced GA over an EPI slice acquisition period (100 ms) averaged over leads and subjects for two different configurations for the standard axial EPI and also for the high-resolution coronal EPI. In Figure 5A, the RMS GA amplitudes were mostly smaller for the modified wiring than the standard wiring at the epochs of highest artifacts (except for the artifact produced by the crusher gradients at ∼55–65 ms). For the axial acquisition, the average RMS of the induced GA showed 11% GA reduction (428 ± 81 μV was reduced to 383 ± 72 μV) after re-wiring in comparison to the standard configuration. It should be noted that the comparatively small alteration in the RMS amplitudes was because of the large duration of the slice TR period (100 ms) where low levels of gradient artifact do not contribute to the RMS. The range of the GA voltages calculated over time and channels shows a comparatively larger difference between the two cap-cable configurations, where the values of range were 12028 ± 1275 and 10504 ± 1716 μV for the standard and modified configurations, respectively.

For the high-resolution coronal EPI acquisition, due to the slice orientation it was expected to have much greater influence of AP gradient on the overall GA, with the largest changes should be induced by the slice select and crusher gradients. This was reflected in the RMS of the artifact over time for coronal acquisition (Figure 5B), which on average was found to be reduced from 1518 ± 102 μV before re-wiring to 753 ± 95 μV after re-wiring, which is equivalent to 50% reduction of the overall induced GA. The greatest advantage of re-wiring the conventional EEG cap was the large reduction of the range of the EEG signal recorded. The average range values over subjects for the coronal acquisition were found to 31709 ± 1056/19957 ± 2028 μV, respectively, for standard/modified wiring. A paired *t*-test of the RMS GA amplitude showed a significant difference (*p* < 0.005) between the standard and modified cap-cable configuration for the axial and coronal acquisitions. In the case of coronal acquisition, two channels were saturated with the standard cable configuration, but there was no channel saturation observed after rewiring (Table 5).

**TABLE 5:** Number of subjects in which channel saturation occurred in standard/modified wiring configuration during coronal acquisition (standard EEG recording bandwidth) in Study 2(ii).

**Channel Name/No**		**Standard ribbon cable**	**Standard twisted cable**	**Modified ribbon cable**	**Channel Name/No.**		**Standard ribbon cable**	**Standard twisted cable**	**Modified ribbon cable**
Fpl	1	0	0	0	Fz	17	0	1	0
Fp2	2	0	0	0	Cz	18	0	0	0
F3	3	0	0	0	Pz	19	0	0	0
F4	4	0	0	0	Oz	20	3	0	2
C3	5	0	0	0	FC1	21	5	5	0
C4	6	0	0	0	FC2	22	1	0	0
P3	7	0	0	0	CP1	23	6	1	5
P4	8	0	0	0	CP2	24	2	2	0
O1	9	0	0	0	FC5	25	6	6	6
O2	10	0	0	0	FC6	26	1	1	1
F7	11	0	0	0	CP5	27	6	0	6
F8	12	0	0	0	CP6	28	2	1	0
T7	13	0	0	0	TP9	29	6	1	6
T8	14	0	0	0	TP10	30	1	2	6
P7	15	0	0	0	POz	31	6	0	6
P8	16	0	0	0	IO	32	6	1	6

In study 2(iii), while the EPI readout gradient was applied in the AP direction and phase-encoding in the RL direction, EEG signal were acquired with higher acquisition bandwidth for the axial EPI acquisition. Twenty-seven (27) scalp channels were saturated for at least one subject (Table 6) with the standard wiring, while fifteen (15) of these were saturated for all subjects and twenty-one (21) were saturated for at least half of the subjects. In contrast, only twenty-one (21) channels were saturated for at least one subject with the modified wiring and among those, only seven channels were saturated for more than half of the subjects. In this study, we could not completely highlight the benefit of re-wiring due to the limitation of the BrainAmp EEG amplifier. EEG amplifier can operate only either at 0.016 – 250 Hz or 0.016 – 1000 Hz; it cannot operate in any frequency between 250 and 1000 Hz. If it could operate up to 500 Hz, we could demonstrate acquisition of EEG signal at higher bandwidth than standard bandwidth (0.016–250 Hz) without saturating any EEG channels using the modified configuration, which is not currently possible with the standard configuration.

**TABLE 6:** Number of subjects in which channel saturation occurred in standard/modified wiring configuration, during axial acquisition with higher (0.016–1000 Hz) EEG recording bandwidth in Study 2(iii).

**Channel Name/No.**		**Standard ribbon cable**	**Standard twisted cable**	**Modified ribbon cable**	**Channel Name/No.**		**Standard ribbon cable**	**Standard twisted cable**	**Modified ribbon cable**
Fpl	1	6	5	3	Fz	17	0	1	0
Fp2	2	6	0	6	Cz	18	0	0	0
F3	3	6	2	2	Pz	19	0	0	0
F4	4	6	1	6	Oz	20	3	0	2
C3	5	6	2	0	FC1	21	5	5	0
C4	6	6	1	6	FC2	22	1	0	0
P3	7	2	0	0	CP1	23	6	1	5
P4	8	6	2	6	CP2	24	2	2	0
O1	9	0	0	2	FC5	25	6	6	6
O2	10	5	5	5	FC6	26	1	1	1
F7	11	0	5	0	CP5	27	6	0	6
F8	12	6	4	6	CP6	28	2	1	0
T7	13	5	2	6	TP9	29	6	1	6
T8	14	6	5	6	TP10	30	1	2	6
P7	15	5	0	6	POz	31	6	0	6
P8	16	6	0	6	IO	32	6	1	6

The variations for the motion parameters were not significant (*p* < 0.005) for the experiments using different configurations. The maximum RMS displacement over the acquisitions was recorded to be less than 1 mm for z-direction translation and less than 0.01° for pitch rotation. It can be noted that this amount of involuntary movement is typically common in any EEG-fMRI experiments.

## Discussion

It was found that the induced GA for the three orthogonal gradients are varied in a complex pattern with the channel number due to the cable connecting the EEG cap and the amplifier (Chowdhury et al., 2015). Previous simulations and experimental work (Yan et al., 2009; Chowdhury et al., 2015) showed that the effect on induced GA from the EEG cap is larger than the ribbon cable. However, the effect from the ribbon cable was substantial particularly when the AP gradient was applied. Figure 1A showed the gradient artifact amplitude on the ribbon cable for different leads while Figure 1B shows the GA from the EEG cap (plus twisted cable). Figure 3 showed how the artifacts from the ribbon cable map onto the scalp along with the channel number and electrode position. AP gradient generated the largest artifact voltages, and the large positive voltages were mapped to the anterior of the head as the channels 1–4 were connected to frontal electrodes (Fp1, Fp2, F3, and F4, respectively), while the large negative voltages were more dispersed over central and lateral regions of the head as channels 27–31 were connected to a range of temporal, parietal and occipital electrodes (CP5, CP6, TP9, TP10, and POz, respectively). Although the GA induced due to the ribbon cable was smaller than that arising from the EEG cap, the addition of the ribbon cable contribution with EEG cap contribution can substantially change the overall spatial distribution of the GA.

Experimental studies were therefore required to validate the assumption of optimal EEG cap-cable configuration which can reduce the overall induced GA. As shown in Study 1, modification of the cap-cable configuration had greatly influenced the magnitude of the induced GAs. It can be noticed from Figure 4A that there was a noteworthy reduction in the GA due to AP gradient, a small reduction for FH gradient and no reduction due to RL gradient were observed with the modified cap-cable configuration. Figure 4B shows that the optimal configuration designed based on the AP gradient contribution from cap and ribbon cable minimized the range of GA for all three gradients. However, the RMS of the artifact over leads was not reduced for the RL gradient.

The results shown in Figures 3B,E for Study 1 clearly depict the superior performance for AP gradient. However, the spatial map (Figures 3A,D) showed that the overall GA contribution from the RL gradient before/after modification is relatively unchanged. This can be explained by the reduction of the artifacts on some channels, whereas the increase in the other channels which did not help in reducing the overall RMS amplitude of the artifact. Since the optimal configuration was designed only keeping the AP gradient in consideration, it is clear that the modifications either reduce or increase the GA amplitude for the other two gradients (RL and FH). However, the above discussion shows that the optimal configuration did not increase the GA contribution for any of the gradients and significantly (*p* < 0.0005) decreased the AP contribution. Moreover, the optimal design produces reduction in range of the GA amplitude for all three gradients, which is extremely advantageous for EEG recording in simultaneous EEG-fMRI experiments. This strategy therefore needed to be evaluated further for the standard EPI studies used in conventional EEG-fMRI experiments, which was the focus of Study 2.

The results of the Study 2 validated the effect of the cap-cabling configurations on the induced GA during the different EPI sequences. It highlighted the dependency of the image geometry, which influenced the temporal features of the GA based on the direction of the applied gradient. The strongest components of the induced GAs were due to the slice select, pre-excursion and crusher gradient pulses for the standard EPI with the EEG recording bandwidth of 250 Hz (Chowdhury, 2014). In order to avoid the image distortions that can disturb the left-right symmetry of the brain, due to field inhomogeneity, axial slice geometry was employed for fMRI data acquisitions and the phase-encoding direction was generally applied in AP. In Study 2(i), the slice-select and phase-encode pre-excursion pulses were applied in the FH and AP directions, respectively. In order to maximize signal dephasing, the crusher gradient pulses generally deployed at all three orthogonal gradients.

Since Study 1 showed that a reduction in the induced GA RMS for the human head could be achieved with the modified configuration for the AP and FH gradients (Figure 4), it was expected that the modified configuration would decrease the overall GA induced by the EPI sequence employed in Study 2(i). The experimental finding was in-line with this expectation and the RMS of the induced GA were reduced from 428 ± 81 μV to 383 ± 72 μV for the modified configuration. As discussed above, the optimal configuration showed improved performance over the standard configuration for the slice-select and pre-excursion gradients. However, in the case of crusher gradients when all three orthogonal gradients were employed simultaneously, there was an increase in the RMS GA amplitude was observed and this might be due to the unequal contribution of different gradients during crusher pulse with the RL gradient contribution might be higher than others.

On the other hand, in Study 2(ii), for high-resolution coronal fMRI data acquisitions, which uses coronal slice and the direction of phase-encoding was chosen to be RL. Therefore, in Study 2(ii), the slice-select and the phase-encode pre-excursion pulses were applied in the AP and RL directions, respectively. The crusher gradient was applied in all three directions. From Study 1, it was expected that coronal slice acquisition will greatly reduce the overall RMS/range of GA amplitude and this was found to be the case. Figure 5B clearly depicts the superior performance of the optimal configuration at the peak artifact pulses. There was an overall 50% reduction of the induced GA and the RMS of the GA was reduced from 1518 ± 102 μV to 753 ± 95 μV after re-wiring. One prominent advantage of re-wiring is found to be the large reduction of the range of the EEG signal recorded (31709 ± 1056 vs. 19957 ± 2028 μV). It should also be noted that using the optimal configuration high-resolution (1.5 mm isotropic resolution) coronal image acquisition can be possible without saturating the EEG amplifier (recording with standard recording bandwidth 0.016–250 Hz); however, this is not possible with the standard configuration (Table 4).

In Study 2(iii), an attempt has been made to evaluate the performance of the modified configuration in comparison to standard configuration in recording EEG data at higher EEG recording bandwidth. However, fMRI data were acquired with the readout gradient in the AP direction and phase-encoding in the RL direction. EEG data were recorded at 0.016–1000 Hz during the acquisition of axial EPI acquisition (course images were acquired to reduce the chance of channel saturation of EEG amplifier). It has been shown in Table 6 that twenty-one channels were saturated in the standard configuration acquisition for most of the subjects, whereas only seven channels were saturated with modified configuration. Brain products amplifier doesn’t allow to record EEG signal at a bandwidth higher than 250 Hz but smaller than 1000 Hz; otherwise, it might be possible to record EEG signal (higher than 250 Hz bandwidth) without saturating any channel using the optimal configuration which is currently not possible with the standard configuration. This could provide an opportunity in the future to reduce the range of the amplifier (currently ± 16.384 mV) required when acquiring in the 0.016–250 Hz bandwidth such that the resolution of the data could then be improved from the current 0.5 μV which would be advantageous for measuring small amplitude neuronal oscillations such as those in the gamma band. Alternatively, the bandwidth over which data may be acquired could be increased. It is clear from Study 2(iii) that a 1000 Hz low–pass filtering saturated some of channels even with the modified cap-cable set-up. However, it may be possible to increase the low-pass filter cut-off frequency into the range 500–750 Hz without any saturation, allowing ultra-high frequency neuronal signals to be recorded (Freyer et al., 2009) without the need for customized MRI sequences. This possibility could not be tested here as the BrainAmp MRplus amplifier can only be set to have a high-frequency cut off of either 250 Hz or 1 kHz. However, it could be envisaged in the future if the EEG amplifier hardware filter is modified by the manufacturer.

Recently Multiband (MB) fMRI has shown the potential to overcome the limitations imposed by conventional sparse fMRI sequences. MB acquisition can be employed to shorten repetition times (TR), increase brain coverage for a given TR, or shorten the acquisition time of whole-head fMRI in a sparse fMRI sequence which would lengthen the gradient-free time window in which EEG data can be collected. Sparse MB fMRI acquisitions, therefore, offer great potential for improving EEG data quality during simultaneous acquisitions. However, recent study from our group in simultaneous EEG-fMRI (Uji et al., 2018) have experienced that standard GA and PA correction techniques were required to apply for the EEG data simultaneously acquired with MB EPI and therefore this study potentially could improve EEG recorded during MB EPI.

Above discussion showed that choosing a particular cap-cabling scheme provided a potential gain of reducing overall RMS and range of the GA amplitude with different image orientations (transverse, coronal, or sagittal slices) while different orthogonal gradients dominate the induced GA. In the future, a different cabling scheme can be obtained for other gradients which could potentially allow us to optimally reduce GA for all three gradients. The quality of EEG data acquired during simultaneous EEG-fMRI would further improve, if additional reduction can be achieved. However, future investigation is essential to identify a single cap-cable configuration that minimizes the variation in induced GA in the three gradient directions by considering the sensitivity to head morphology which varies from subject to subject.

## Conclusion

Interference between gradient artifacts induced in the EEG cap and in the cable that connect the EEG cap to the amplifier can be used to minimize the overall range and RMS amplitude of the GA. Here by modifying the connections of the EEG cap and amplifier, we were able to reduce the range of the GA for a high- resolution coronal EPI acquisition by a factor of ∼ 1.6 and by a factor of ∼ 1.15 for a standard axial EPI acquisition. These changes could potentially be translated into a reduction in the required dynamic range, an increase in the EEG bandwidth or an increase in the achievable image resolution without saturation, all of which could be beneficially exploited in EEG-fMRI studies. The re-wiring could also prevent the system from saturating when small subject movements occur using the standard recording bandwidth. Our focus here was on reducing the GA due to the AP gradient, but alternative cabling schemes which additionally reduce the overall GA from RL and FH gradients can also be envisaged and will be explored in the future work.

## Ethics Statement

All experiments were carried out at the Sir Peter Mansfield Imaging Centre (SPMIC), University of Nottingham, Nottingham, United Kingdom. Experiments on human subjects were carried out with the written consent from the subjects and with the approval from local ethical committee (ethical committee of the University of Nottingham).

## Author Contributions

MC, KM, and RB designed the experiments. MC and KM performed the experiments. MC, AK, KM, and RB analyzed the results. All authors were involved in interpretation of the data and wrote the manuscript.

## Conflict of Interest Statement

The authors declare that the research was conducted in the absence of any commercial or financial relationships that could be construed as a potential conflict of interest.
